# Disparities in liver-related care among transgender adults with steatotic liver disease: a retrospective cohort study

**DOI:** 10.1210/jendso/bvag129

**Published:** 2026-06-13

**Authors:** Maria Ortega Abad, Leandro Sierra, Nikki Duong, Ricardo Correa

**Affiliations:** Endocrinology and Metabolism Institute, Cleveland Clinic, 9500 Euclid Avenue, Cleveland, OH 44195, USA; Department of Internal Medicine, Cleveland Clinic, Cleveland, OH 44195, USA; Division of Gastroenterology and Hepatology, Stanford University, Palo Alto, CA 94305, USA; Endocrinology and Metabolism Institute, Cleveland Clinic, 9500 Euclid Avenue, Cleveland, OH 44195, USA

**Keywords:** transgender, steatotic liver disease, metabolic-associated steatotic liver disease, healthcare disparities, gender-affirming care

## Abstract

**Background:**

Transgender (TGD) adults experience healthcare disparities across multiple domains, yet care for steatotic liver disease (SLD) remains uninvestigated.

**Aims:**

Evaluate whether TGD adults with SLD receive equitable liver-related care compared with cisgender (CGD) adults.

**Methods:**

Retrospective cohort study conducted at a tertiary medical center (2015-2025). Adults with SLD were identified by a 2-step process (ICD-10 codes, manual chart review). Patients with other causes of liver disease and missing data were excluded. TGD status was defined by ICD-10 codes for gender dysphoria, documented gender incongruence or use of gender affirming hormone therapy. Controls and cases were selected from the same cohort with a 2:1 sampling ratio. Primary outcomes, gastroenterology consultation, hepatology assessment (FIB-4, elastography, or biopsy), and advanced workup, were analyzed using logistic regression adjusting for age and cardiometabolic covariates. An age restricted (30-60) sensitivity analysis was performed. Secondary outcomes included viral hepatitis screening/vaccination, SLD-directed therapies, and 5-year BMI trajectories.

**Results:**

Among 254 adults (85 TGD, 169 CGD), TGD individuals were younger (36.0 vs 57.0 years, *P* < .001) with higher MASLD prevalence (91.8% vs 77.5%, *P* = .05). TGD status was associated with lower odds of gastroenterology consultation (aOR 0.41; 95% CI .18–.93), any hepatology assessment (aOR 0.35; 95% CI .18–.70), advanced hepatology workup (aOR 0.34; 95% CI .17–.69), with robust findings in sensitivity analysis. GLP-1 agonist prescriptions were lower (9.4% vs 19.5%, *P* = .03), and BMI increased vs controls (*P* < .001).

**Conclusion:**

TGD adults with SLD experience substantial disparities in care, requiring integrated, inclusive pathways to advance health equity.

Steatotic liver disease (SLD) is among the most prevalent chronic liver conditions worldwide. Metabolic dysfunction-associated steatotic liver disease (MASLD), the predominant subtype, affects approximately 30% of adults, with a rising prevalence among younger populations [1]. The growing burden of obesity, type 2 diabetes, and metabolic syndrome, driven by insulin resistance, visceral adiposity, and dyslipidemia, has contributed to a parallel rise in SLD [2, 3]. Risk stratification using FIB-4 score calculation and liver elastography is a cornerstone of SLD management, yet implementation remains inconsistent across clinical settings [4, 5].

Among populations affected by these care gaps, transgender (TGD) individuals merit particular attention, comprising an estimated 0.8% of the United States adult population with rising prevalence among younger cohorts [6]. This population experiences well-documented healthcare disparities, including reduced access to preventive services, cancer screening, and specialty care [7-9]. Compared with CGD peers, TGD adults have 53-98% lower odds of receiving recommended breast and cervical cancer screening and 76-80% lower odds of colorectal cancer screening [10, 11].

In addition, several factors suggest that TGD individuals may face elevated SLD risk. Gender-affirming hormone therapy (GAHT) influences hepatic metabolism and may alter transaminase levels [12, 13]. Testosterone therapy in transgender men has been associated with higher risk of hepatic steatosis, whereas the metabolic effects of estrogen therapy in transgender women remain incompletely characterized [14]. Furthermore, TGD adults experience higher rates of depression and anxiety—conditions independently associated with increased MASLD risk through behavioral and biological pathways [15].

Despite this evidence and growing attention to TGD-related disparities across other medical specialties, hepatology care patterns among TGD adults with SLD remain largely unexplored [16]. This knowledge gap is pressing, given the rising prevalence of both MASLD and TGD individuals in the general population. Therefore, we conducted a retrospective cohort study comparing liver-related care between TGD and CGD adults with SLD at a large academic medical center.

## Methods

### Study design and setting

This retrospective cohort study was conducted at Cleveland Clinic (Cleveland, Ohio) between January 1, 2015, and December 31, 2025. The study was approved by the Cleveland Clinic Institutional Review Board with a waiver of informed consent.

### Participants

**Figure 1:**
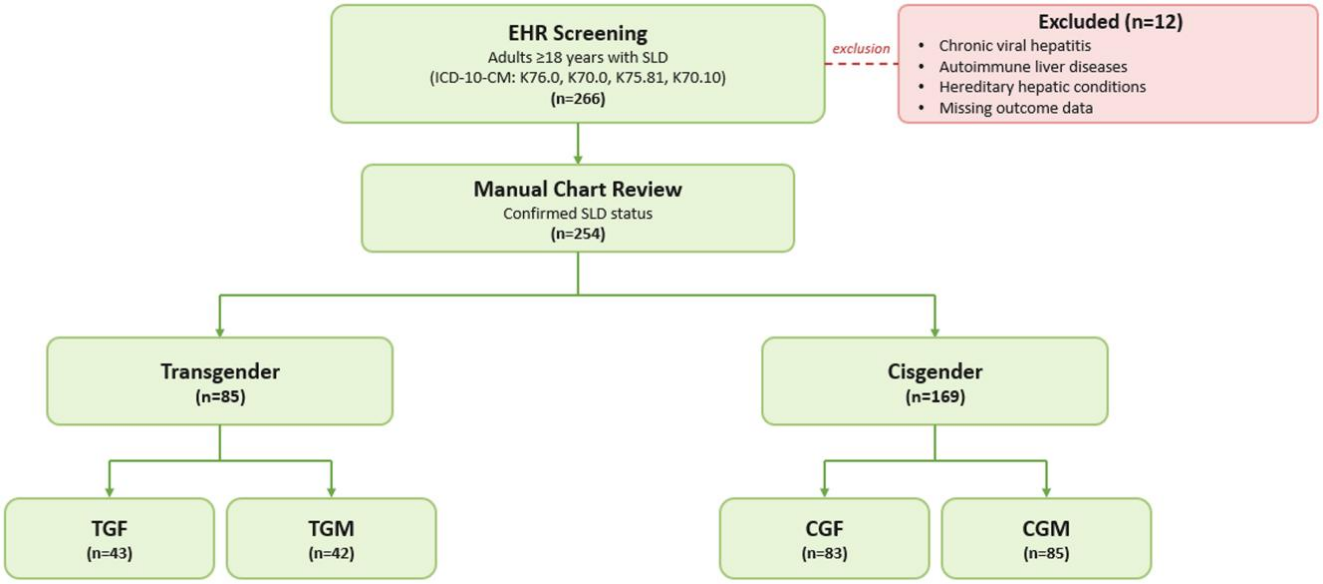
Patient selection flowchart. Electronic health record screening identified adults with steatotic liver disease using ICD-10-CM codes. Manual chart review confirmed diagnoses. Twelve patients were excluded due to competing liver disease etiologies or missing outcome data. CGD controls were from the same SLD population. CGD, cisgender, CGF, cisgender female; CGM, cisgender male; EHR, electronic health record; FTM, transgender male; MTF, transgender female; SLD, steatotic liver disease; TGD, transgender and gender-diverse.

Adults aged ≥18 years with confirmed SLD were eligible. A 2-stage identification process ensured diagnostic accuracy: (1) electronic health record screening using ICD-10-CM codes for SLD (K76.0 [fatty liver, not elsewhere classified], K70.0 [alcoholic fatty liver], K75.81 [nonalcoholic steatohepatitis], K70.10 [alcoholic hepatitis]); (2) manual chart review to confirm SLD diagnosis based on imaging findings (hepatic steatosis on ultrasound, CT, or MRI), laboratory values (elevated transaminases), or histopathology. Patients with competing etiologies of liver disease, including chronic viral hepatitis, autoimmune hepatitis, and hereditary liver disease, and those with missing data for outcome variables were excluded (Fig. 1).

TGD status was defined by ICD-10 codes (F64.0 [transsexualism], F64.9 [gender disorder, unspecified]), documented gender identity discordant with sex assigned at birth, or receipt of GAHT. TGD cases and CGD controls were selected from the same SLD cohort. A control-to-case sampling ratio of 2:1 was used to improve statistical power due to the uncommon prevalence of TGD status [17].

### Measures of exposure and covariates

The primary exposure of interest in this study was TGD status, subcategorized as transgender female (MTF; male sex assigned at birth, female gender identity) or transgender male (FTM; female sex assigned at birth, male gender identity). Demographic and clinical covariates included: age, race, body mass index (BMI), cardiometabolic and psychiatric comorbidities, GAHT regimens (estrogen-based with or without progesterone and/or antiandrogens for MTF individuals and testosterone-based for FTM individuals), and serum sex hormone concentrations. SLD was subclassified according to 2023 American Association for the Study of Liver Diseases (AASLD) nomenclature as MASLD, metabolic dysfunction and alcohol-associated liver disease (MetALD), or alcohol-associated liver disease (ALD) [4].

### Outcomes

The primary outcome was liver-related care, classified into 3 ordered levels reflecting escalation of care: (1) gastroenterology consultation, defined as any documented outpatient or inpatient gastroenterology or hepatology encounter; (2) any hepatology assessment, defined as documentation of FIB-4 calculation, transient elastography (FibroScan), or liver biopsy; and (3) advanced workup, defined as completion of transient elastography or liver biopsy in conjunction with gastroenterology/hepatology consultation. Secondary outcomes included hepatitis screening and vaccination, receipt of SLD-directed therapeutics (vitamin E, glucagon-like peptide-1 receptor agonists [GLP-1 RAs], sodium-glucose cotransporter-2 inhibitors [SGLT2i], resmetirom, bariatric surgery), and BMI trajectories from the initial gastroenterology visit through 5 years of follow-up.

### Statistical analysis

Continuous variables are presented as mean ± standard deviation (SD) and compared using Student's t-test or Mann-Whitney U test as appropriate. Categorical variables are presented as frequencies (percentages) and compared using chi-square or Fisher's exact tests. Time intervals from initial gastroenterology consultation to liver fibrosis assessment (FIB-4 calculation, Fibroscan, or liver biopsy), vaccination (HAV and HBV), and therapy (first dose of GLP-1, bariatric surgery) were compared between groups using Kruskal-Wallis. For the primary analysis, we employed Firth's penalized logistic regression to estimate adjusted odds ratios (aOR) with 95% confidence intervals, adjusting for age, diabetes mellitus, hypertension, dyslipidemia, BMI at diagnosis, and alcohol use disorder. Firth's method was selected to address potential sparse data bias given the modest sample size. To assess the robustness of our findings and account for age differences between groups, we conducted a sensitivity analysis restricted to participants aged 30—60 years, corresponding to the overlapping age range between the TGD and CGD groups. Longitudinal BMI trajectories were compared using linear mixed-effects models, from initial gastroenterology visit to last BMI recorded within 5 years. Subgroup analyses compared outcomes across 4 groups: MTF, FTM, cisgender female (CGF), and cisgender male (CGM). Statistical significance was defined as 2-sided *P* < .05. Analyses were performed using Stata (v19; StataCorp).

## Results

### Study population

The final cohort included 254 adults: 85 TGD (43 MTF, 42 FTM) and 169 CGD controls (85 CGF, 84 CGM). Baseline characteristics are presented in Table 1. TGD participants were significantly younger (mean age 36.0 ± 13.2 vs 57.0 ± 14.1 years, *P* < .001) and had higher MASLD prevalence (91.8% vs 77.5%, *P* = .05) compared with controls. However, MetALD was more common in the CGD group (12.4% vs 3.5%, *P* = .02). ALD was similar between groups. Cardiometabolic comorbidities were lower, while psychiatric comorbidities were higher in the TGD group compared with controls (type 2 diabetes mellitus 38.8% vs 53.3%, *P* = .03; hypertension 31.8% vs 58%, *P* < .01; depression 58.8% vs 32.1%, *P* < .01; generalized anxiety disorder 57.6% vs 14.2%, *P* < .01; bipolar disorder 20% vs 5.9%, *P* < .01).

**Table 1:** Baseline characteristics and steatotic liver disease care in transgender versus cisgender patients

Variable	Transgender (*n* = 85)	Cisgender (*n* = 169)	*P*-value
**Demographics**			
Age, years*^a^*	36 ± 13.2	57 ± 14.1	**<**.**01**
BMI, kg/m^2^*^a^*	36.1 ± 8.4	34.1 ± 8.4	.07
Race, White	69 (81.2)	128 (75.7)	.58
**Comorbidities**			
Diabetes	33 (38.8)	90 (53.3)	.**03**
OSA	24 (28.2)	47 (27.8)	.94
Hypertension	27 (31.8)	98 (58.0)	**<**.**01**
Dyslipidemia	31 (36.5)	72 (42.6)	.48
Depression	50 (58.8)	39 (23.1)	**<**.**01**
Generalized anxiety disorder	49 (57.6)	24 (14.2)	**<**.**01**
Bipolar disorder	17 (20.0)	10 (5.9)	**<**.**01**
**SLD Etiology**			
MASLD	78 (91.8)	131 (77.5)	**<**.**01**
MetALD	3 (3.5)	21 (12.4)	.**02**
ALD	4 (4.7)	17 (10.1)	.14
**Hepatology Care**			
Gastroenterology consultation	61 (71.8)	141 (83.4)	.**03**
FIB-4 documentation	7 (8.2)	12 (7.1)	.75
Liver elastography	19 (22.4)	83 (49.1)	**<**.**01**
Liver biopsy	9 (10.6)	52 (30.8)	**<**.**01**
**Viral hepatitis preventive care**			
HAV vaccination	27 (31.8)	29 (17.2)	.**01**
HBV vaccination	32 (37.7)	36 (21.3)	**<**.**01**
HAV screening	31 (36.5)	85 (50.3)	.**04**
HBV screening	54 (63.5)	123 (78.2)	.14
HCV screening	70 (82.4)	141 (83.4)	.86
**Therapeutics**			
Vitamin E	0 (0.0)	7 (4.1)	.06
GLP-1 RA	8 (9.4)	33 (19.5)	.**03**
SGLT2i	1 (1.2)	10 (5.9)	.08
Resmetirom	1 (1.2)	1 (0.6)	.62
Bariatric surgery	6 (7.1)	15 (8.9)	.62

Data presented as *n* (%) unless otherwise indicated, bold values indicate statistical significance.

^*a*^Mean ± SD.

Abbreviations: ALD, alcohol-associated liver disease; BMI, body mass index; FIB-4, Fibrosis-4 index; GLP-1 RA, glucagon-like peptide-1 receptor agonist; HAV, hepatitis A virus; HBV, hepatitis B virus; HCV, hepatitis C virus; MASLD, metabolic dysfunction-associated steatotic liver disease; MetALD, metabolic dysfunction and alcohol-associated liver disease; OSA, obstructive sleep apnea; SGLT2i, sodium-glucose cotransporter-2 inhibitor; SLD, steatotic liver disease.

### Gender-affirming hormone therapy and sex hormone levels

Among TGD participants, 88.2% received GAHT. MTF adults received combined estrogen, progesterone, and antiandrogen therapy more frequently (39.5%), while FTM individuals predominantly received testosterone therapy (88.1%). Serum hormone concentrations reflected GAHT regimens (Table 2). Median serum testosterone was highest in FTM (381 ng/dL [IQR 154-576]; 13.2 nmol/L [5.3-20.0]), followed by CGM (279 ng/dL [215-528]; 9.7 nmol/L [7.5-18.3]), CGF (23 ng/dL [12-35]; 0.80 nmol/L [0.42-1.2]), and MTF (21.5 ng/dL [13-45]; 0.75 nmol/L [0.45-1.56]) (*P* < .001). Median serum estradiol was highest in MTF (145 pg/mL [86-303]; 532 pmol/L [316-1112]), followed by CGF (41.5 pg/mL [27-50]; 152 pmol/L [99-184]) and FTM (29 pg/mL [20-48]; 106 pmol/L [73-176]) (*P* < .001).

**Table 2:** Baseline characteristics and steatotic liver disease care by gender identity

Variable	MTF	FTM	CGF	CGM	*P*-values				
					MTF vs FTM	MTF vs CGM	MTF vs CGF	FTM vs CGF	FTM vs CGM
n	43	42	83	85					
**Demographics**									
Age, years*^a^*	39.9 ± 14	31.4 ± 11	56.9 ± 14	57.3 ± 14	**<**.**01**	**<**.**01**	**<**.**01**	**<**.**01**	**<**.**01**
BMI, kg/m^2^*^a^*	35.8 ± 8.8	36.6 ± 8.2	35.7 ± 8.5	32.6 ± 8.0	.62	.**05**	.98	.55	.**01**
**Comorbidities**									
Diabetes	17 (39.5)	16 (38.1)	45 (54.2)	45 (52.9)	.89	.15	.13	.10	.12
OSA	14 (32.6)	10 (23.8)	24 (28.9)	23 (27.1)	.37	.52	.64	.57	.69
Alcohol use disorder	8 (18.6)	3 (7.1)	19 (22.9)	24 (28.2)	.12	.24	.60	.**03**	.**01**
Hypertension	20 (46.5)	7 (16.7)	52 (62.7)	45 (52.9)	**<**.**01**	.49	.07	**<**.**01**	**<**.**01**
Dyslipidemia	17 (39.5)	14 (33.3)	37 (44.6)	35 (41.2)	.55	.75	.63	.25	.52
Depression	26 (60.5)	24 (57.1)	20 (24.1)	19 (22.4)	.76	**<**.**01**	**<**.**01**	**<**.**01**	**<**.**01**
Anxiety disorder	24 (55.8)	25 (59.5)	11 (13.3)	13 (15.3)	.73	**<**.**01**	**<**.**01**	**<**.**01**	**<**.**01**
Bipolar disorder	7 (16.3)	10 (23.8)	6 (7.2)	4 (4.7)	.39	.**03**	.11	.**01**	**<**.**01**
**SLD Subclassification**									
MASLD	39 (90.7)	39 (92.9)	66 (78.6)	65 (76.0)	.72	.**05**	.09	.**04**	.**02**
MetALD	1 (2.3)	1 (2.4)	7 (8.3)	10 (11.8)	.25	.07	.**05**	.15	.20
ALD	3 (7.0)	3 (7.1)	19 (22.9)	20 (23.5)	.54	.40	.79	.20	.08
**Serum sex-hormone levels*^b^***									
Estradiol- 17β, pg/dL [pmol/L]	145 (86-303) [532 (316-1112)]	29 (20-288); [106 (73-1055)]	41.5 (27-50); [152 (99-184)]	—	**<**.**01**	—	.35	**<**.**01**	—
Testosterone, ng/dL [nmol/L]	21.5 (13-47) [0.75 (0.45-1.63)]	381 (154-576) [13.2 (5.3-20.0)]	23 (12-55) [0.80 (0.42-1.91)]	279 (215-528) [9.7 (7.5-18.3)]	**<**.**01**	**<**.**01**	.70	**<**.**01**	.42
**Hepatology Care**									
Gastroenterology consultation	31 (72.1)	30 (71.4)	74 (88.1)	67 (78.8)	.95	.40	.**02**	.**02**	.36
Liver elastography	10 (23.3)	9 (21.4)	43 (51.2)	29 (47.1)	.84	.**01**	**<**.**01**	**<**.**01**	.**01**
Liver biopsy	5 (11.6)	4 (9.5)	23 (27.4)	29 (34.1)	.78	.**01**	.**04**	.**02**	**<**.**01**
HAV vaccination	18 (41.9)	9 (21.4)	17 (20.2)	12 (14.1)	.**04**	**<**.**01**	.**01**	.87	.29
HBV vaccination	22 (51.2)	12 (28.6)	22 (26.2)	15 (17.6)	.**03**	**<**.**01**	**<**.**01**	.77	.15
HAV screening	17 (39.5)	14 (33.3)	36 (42.9)	49 (57.6)	.55	.05	.71	.30	.**01**
HBV screening	30 (69.8)	24 (57.1)	62 (73.8)	61 (71.8)	.22	.81	.62	.05	.09
HCV screening	39 (90.7)	31 (73.8)	72 (85.7)	69 (81.2)	.**04**	.16	.42	.10	.34

Data presented as *n* (%) unless otherwise indicated, bold values indicate statistical significance.

^*a*^Mean ± SD.

^*b*^Median (IQR).

(−) no data recorded.

Abbreviations: ALD, alcohol-associated liver disease; BMI, body mass index; CGF, cisgender female; CGM, cisgender male; FTM, transgender male; HAV, hepatitis A virus; HBV, hepatitis B virus; HCV, hepatitis C virus; IQR, interquartile range; MASLD, metabolic dysfunction-associated steatotic liver disease; MetALD, metabolic dysfunction and alcohol-associated liver disease; MTF, transgender female; OSA, obstructive sleep apnea; SLD, steatotic liver disease.

### Liver-related assessment

**Figure 2:**
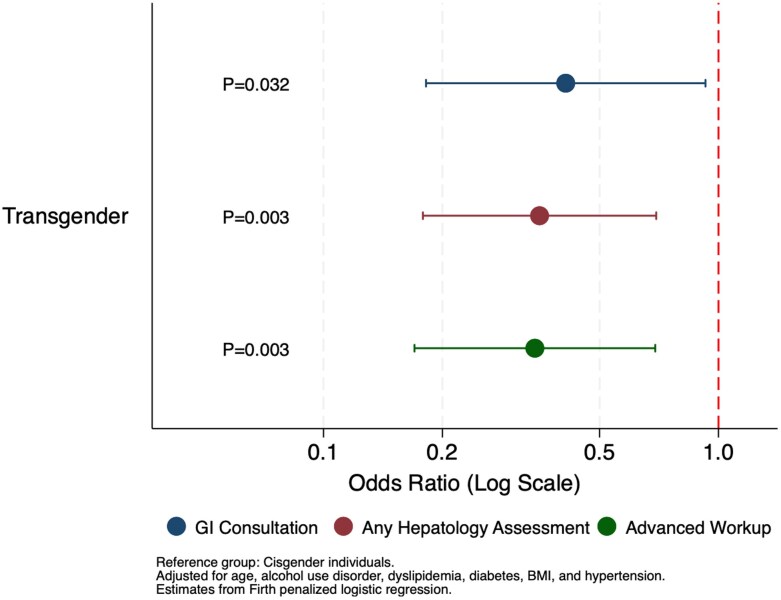
Adjusted odds ratios for steatotic liver disease care outcomes in transgender versus cisgender patients. Forest plot depicting adjusted odds ratios (aORs) with 95% confidence intervals for steatotic liver disease care outcomes comparing transgender to cisgender patients. The vertical dashed red line indicates an odds ratio of 1.0 (no difference). All point estimates fall to the left of 1.0, indicating that transgender patients had significantly lower odds of receiving each SLD care outcome compared with cisgender controls. Models were adjusted for age, type 2 diabetes, hypertension, dyslipidemia, baseline BMI, and alcohol use disorder. Estimates were derived from Firth penalized logistic regression to account for sparse data. aOR, adjusted odds ratio; CI, confidence interval; GI, gastroenterology; SLD, steatotic liver disease.

TGD adults had lower rates of gastroenterology consultation (71.8% vs 83.4%; *P* = .03), liver elastography (22.4% vs 49.1%; *P* < .01), and liver biopsy (10.6% vs 30.8%; *P* < .01). FIB-4 documentation rates were low in both groups (8.2% vs 7.1%). After adjustment for age, diabetes, hypertension, dyslipidemia, BMI at diagnosis, and alcohol use disorder (Fig. 2), TGD status remained significantly associated with reduced odds of receiving care across all defined levels (gastroenterology consultation [aOR 0.41; 95% CI .18–.93; *P* = .03], any hepatology assessment [aOR 0.35; 95% CI .18–.70; *P* = .003], and advanced hepatology workup [aOR 0.34; 95% CI .17–.69; *P* = .003]). There were no significant differences in time from the initial gastroenterology visit to the first hepatology assessment between transgender and CGD patients (19.4 months [IQR, 1.7-32.2] vs 10.8 months [IQR, 1.5-38.8], respectively; *P* = .81).

### Viral hepatitis preventive care

Overall, HAV and HBV vaccination rates were significantly higher among TGD individuals compared with CGD individuals (31.8% vs 17.2%, *P* = .01; 37.7% vs 21.3%, *P* < .01, respectively) (Table 1). Among patients requiring vaccination based on serologic immune status, HBV vaccine completion rates were higher in TGD individuals (58.3% vs 34.5%; *P* = .04), whereas HAV vaccine completion rates were similar between groups (37.5% vs 27.3%; *P* = .58). Time from initial gastroenterology consultation to hepatitis vaccination did not differ significantly (TGD: 33.2 months [IQR 9.1-87.8] vs CGD: 8.1 months [IQR 1.8-43.3]; *P* = .23). Regarding hepatitis screening, HAV testing was less frequently performed in TGD individuals (36.5% vs 50.3%, *P* = .04), while HBV and HCV screening rates were similar between groups (63.5% vs 78.2%, *P* = .14; 82.4% vs 83.4%, *P* = .86, respectively).

### SLD-targeted therapies

**Figure 3:**
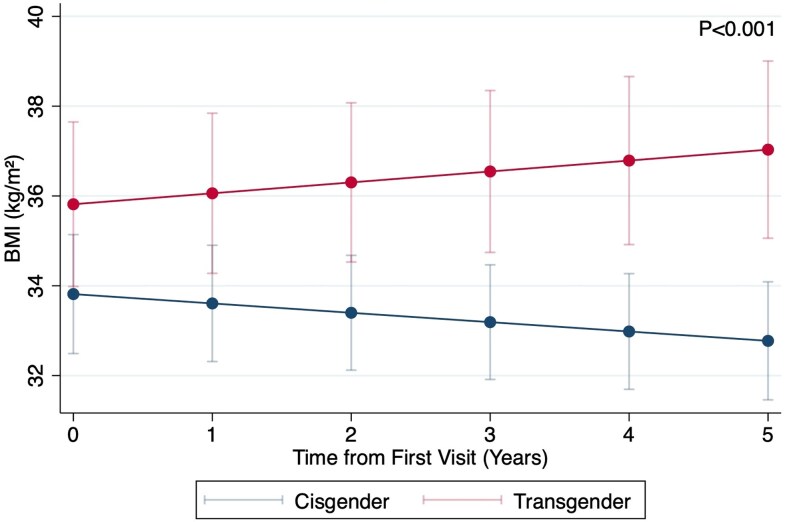
Body mass index trajectories over 5 years. Mean BMI with 95% CI stratified by TGD and CGD status. TGD individuals demonstrated progressive weight gain, whereas cisgender individuals showed modest weight reduction over the study period (*P* < .001). BMI, body mass index; CGD, cisgender; CI, confidence interval; TGD, transgender.

Regarding therapy, GLP-1 RA prescription was lower among TGD individuals (9.4% vs 19.5%; *P* = .03). Use of vitamin E, SGLT2 inhibitors, resmetirom, and bariatric surgery was infrequent in both groups. Among patients eligible for weight management therapy, GLP-1 RA prescription rates were significantly lower in TGD compared with CGD individuals (10.7% vs 25.4%; *P* = .01), whereas bariatric surgery rates were similar (12.2% vs 16.9%; *P* = .50). Time to first GLP-1 RA dose did not differ significantly between groups (22.7 months [IQR 18.6-33.9] vs 55.0 months [IQR 26.7-87.6]; *P* = .08). BMI trajectories diverged significantly over 5 years (*P* < .001; Fig. 3): TGD individuals demonstrated progressive weight gain (35.9 to 37.0 kg/m^2^), whereas CGD individuals showed modest weight reduction (33.9 to 32.7 kg/m^2^).

### Subgroup comparisons

Four group comparisons demonstrated nuanced differences (Table 2). MTF adults were significantly older than FTM (39.9 ± 14 vs 31.4 ± 11 years; *P* = .003) and had higher hypertension prevalence at baseline (46.5% vs 16.7%; *P* = .03). CGM showed lower BMI compared with MTF and FTM (*P* = .046 and *P* = .009, respectively). Mental health disorders, including depression and anxiety, were more common in both MTF and FTM compared with CGD controls (all *P* < .05). Regarding SLD subclassification, FTM demonstrated a higher prevalence of MASLD relative to CGF and CGM (92.2% vs 78.6% vs 76.0%; *P* = .04 and *P* = .02, respectively), whereas no etiologic differences emerged between MTF and CGD comparators. MetALD and ALD distributions were similar across all groups. No significant differences in SLD were seen between MTF vs FTM.

Regarding liver-related care, liver elastography completion was significantly lower in both MTF and FTM individuals compared with CGD groups (MTF 23.3%, FTM 21.4% vs CGF 51.2%, CGM 47.1%; all *P* < .01). Similarly, liver biopsy rates were lower among TGD individuals (MTF 11.6%, FTM 9.5% vs CGF 27.4%, CGM 34.1%; all *P* < .05). Conversely, HAV and HBV vaccination rates were highest among MTF individuals (41.9% and 51.2%, respectively), significantly exceeding all other groups (all *P* < .05).

### Sensitivity analysis

We performed a restricted analysis among participants aged 30-60 years (*N* = 137; 54 TGD, 83 CGD). This analysis demonstrated a persistent and statistically significant association between TGD status and lower odds of receiving care across all predefined levels (gastroenterology consultation [aOR 0.28; 95% CI .10–.79; *P* = .01], any hepatology assessment [aOR 0.33; 95% CI .13–.84; *P* = .02], and advanced hepatology workup [aOR 0.27; 95% CI .10–.71; *P* = .008]).

## Discussion

In this retrospective cohort study, we provide the first evidence that TGD adults with SLD experience substantial disparities compared with CGD adults. TGD individuals had 55-65% lower adjusted odds of receiving gastroenterology consultation, hepatology assessment, and advanced fibrosis workup, disparities that persisted after controlling for age and cardiometabolic comorbidities. These findings add to the growing literature on TGD healthcare disparities and carry important implications for clinical practice.

Several aspects of our findings warrant discussion. First, the higher prevalence of MASLD in TGD adults (91.8% vs 77.5%; *P* = .05) despite younger age and lower cardiometabolic burden suggests that nontraditional risk factors may drive SLD in this population. Potential contributors include GAHT effects on hepatic lipid metabolism, higher rates of psychiatric comorbidity, and psychosocial stressors related to minority stress [18, 19].

Preclinical studies demonstrated that female mice exposed to dihydrotestosterone (DHT) develop hepatic steatosis and insulin resistance, unlike male rodents, though it remains unclear whether these effects are direct or mediated by adiposity [20]. Clinically, free testosterone has been linked to metabolic associated-steatohepatitis (MASH) fibrosis in premenopausal CGF, independent of abdominal adiposity and diabetes (OR: 1.97, 95% CI; 1.03-3.79, *P* = .04), supporting a direct hepatic mechanism [14]. In our cohort, FTM had the highest testosterone levels and MASLD prevalence, highlighting the importance of hepatic monitoring in FTM adults receiving testosterone. Yet paradoxically, this group had the lowest rates of hepatology consultation in our study (71.4%).

In addition, the high burden of psychiatric comorbidity in TGD adults may further exacerbate MASLD risk. In our cohort, depression and anxiety rates were nearly twice those of CGD controls, consistent with prior European data [21]. Depression, as well as other psychiatric conditions, is related to chronic inflammation and can induce immune-mediated β-cell injury, leading to insulin resistance: a key driver of MASLD [22]. Mendelian randomization studies have demonstrated that depression increases MASLD risk by 56% (OR 1.56; 95% CI 1.10-2.21), providing biological plausibility for this association [23].

Second, disparities in liver-related care were pronounced despite comparable times from initial gastroenterology consult to first assessment, suggesting that multilevel barriers may operate at the point of referral rather than within the care pathway itself. At the patient level, prior research has documented that approximately one-third of TGD individuals avoid healthcare due to fear of discrimination and prioritize gender-affirming care over other health needs [24]. Medical mistrust stemming from historical mistreatment may further reduce engagement with specialty care.

At the systems level, the reduced awareness of TGD-specific health problems and issues with insurance coverage create structural barriers to equitable care [25]. For instance, the lower rates of GLP-1 RA prescribing and use observed among TGD, along with significantly higher BMI at 5 years, likely reflect underlying insurance-related inequities. In addition, it is worth noting that disparities in healthcare access and insurance coverage between younger and older patients may also influence access to care, as young adults have increased vulnerability to being uninsured compared with older populations [26]. Furthermore, the universally low FIB-4 documentation (7-8%) observed in our study reflects a system-wide failure in fibrosis risk stratification, disproportionately burdening vulnerable populations such as TGD adults. This could be addressed through standardized EHR tools, including dot phrases and automated FIB-4 documentation [27].

Third, we observed divergent patterns in viral hepatitis preventive care. Overall vaccination rates were higher among TGD, driven by MTF individuals who had the highest HAV (41.9%) and HBV (51.2%) vaccination rates across all groups. This finding aligns with previous data and reflects higher predisposition to preventive services access among TGD individuals [28]. However, HAV screening was less frequently performed in TGD (36.5% vs 50.3%; *P* = .04), indicating that vaccination alone does not ensure complete hepatitis care.

This study has several strengths, including its novel focus on an understudied population, rigorous 2-stage case identification with manual chart verification, and use of Firth's penalized regression to address sparse data. The hierarchical outcome framework provides a comprehensive assessment of liver-related care across multiple domains. Although, limitations must be acknowledged. The retrospective single-center design limits generalizability and precludes causal inference. The significant age difference between TGD and CGD cohorts represents a key limitation; although age was included as a continuous covariate in all multivariable models, residual confounding cannot be fully excluded with a 21-year mean age gap. However, our sensitivity analysis restricted to participants aged 30-60 years revealed consistent disparities after adjustment for age, AUD, dyslipidemia, diabetes, BMI, and hypertension. Differences in GLP-1 RA prescribing should be interpreted with caution, as the CGD group had a higher prevalence of diabetes. We lacked longitudinal fibrosis data, precluding assessment of disease progression. Unmeasured variables including insurance status, socioeconomic factors, geographic access, and GAHT duration may influence both TGD status and healthcare utilization. Finally, Subgroup analyses by gender identity subgroup are exploratory and should be interpreted with caution given the modest within-group sample sizes.

Our findings support several clinical recommendations. First, SLD screening should be integrated into routine primary care visits, particularly for individuals receiving testosterone therapy. Second, provider education should address TGD-specific hepatic risks, appropriate monitoring intervals, and timely hepatology referral for fibrosis assessment. Third, promoting the creation of specialized centers for LGBTQ + patients may ensure expert, equitable care is executed. Future research should prioritize prospective cohort studies evaluating SLD progression in TGD populations compared with age- and comorbidity- matched controls; validation of noninvasive fibrosis scores in TGD populations; qualitative and mixed-methods studies to characterize patient- and provider-level barriers; and the development and implementation of quality improvement strategies to reduce gender-related disparities in SLD care.

In conclusion, TGD adults with SLD experience substantial disparities in liver fibrosis assessment, viral hepatitis prevention, and GLP-1 RA therapy. With MASLD prevalence rising globally and TGD individuals comprising a growing proportion of the population, these findings underscore an urgent need for integrated care models, provider education, and inclusive clinical practice guidelines. Addressing SLD care disparities in TGD populations represents both a clinical imperative and an opportunity to advance health equity.

## Data Availability

Some or all datasets generated during and/or analyzed during the current study are not publicly available but are available from the corresponding author on reasonable request.
